# Supplementary material for: Chromothripsis during telomere crisis is independent of NHEJ, and consistent with a replicative origin
Source: Genome Res. 2019 May;29(5):737–49. doi: 10.1101/gr.240705.118 (PMC6499312; doi:10.1101/gr.240705.118)
Supplement: Supplemental Material [file supp_gr.240705.118_Supplemental_file_1.zip › contigs/annotated_contigs/DB49/contig.3.DB49_length_1021_mean_cov_13.8148873653.docx]

**DB49_length_1021_mean_cov_13.8148873653**

```
ATTCACTAATCAGTTGTCAGTTGGCAACTTGATT|GCAAACGAGCCTGACTCACACTGAAGTTATCTGCAATCAAGTTGCCAACTGACA
 >chrY:59018596-59018630 - E=2e-08  >chrUn_gl000225:160585-161216 + E=0e+00 p=0e+00
ACTGATTAGTGAATGAAGCTGAGCGCCAGGACTCACGACAGCTGTCTGTTTCATAAACACGCCCTCGTTCCTCCTTTTGGCCGACTACT

AAAGGGTTTAAGTGTCACGCAGCATTTAGATGTTATGCAGAATGAGCAACAAGCAAAATGTAGGTGCAATCAGATAAGAGTGGAGAATT

ACCAGTATCTTTACAATCACCTGTTGGGCATTTCTGTTGATAATCACCAATTCAGAACAGCAAGGACTGAGAAGCCACAAGATTCCCTT

AAAAGGCCTCATCCACTTGCATACTTTTGTTTTCAGGAAACAAAAATTATGTTATGTGGGCTCACAGTCAATAGATTTTTGTTCTGATT

TGTATTTCCATGATACATAATTAAGTTTTGTGGTTTGAGGTTGAAAGAATTCTGAATGTGTATGCTAACACTGATTCACATTAAAGCTA

AGCACTAGGCTGGACACGATGGCTTACGCCTGTAATCCCAACACTTTGGGAGGCCGAGGCGGACGGATCACTTGAGGTCAGGAGTTTGA

GACCAGCCTGGCCAACATGGTGAAACCCCATCTCTACTAAAAA|TACAAAAAAAAAAATTA|GCCAGGCGTGGTGGCACGTACCTGTAG
                                                             >chr5:31905952-31906291 -
TCCCAGCTACTCGGGAGGCTGAGGCGGGAGAATGACATGAACCCGGGAGGCAGAGCTTGTAGTAAGCCGAGACGGTGCCACTGCACTCC
E=7e-183
AGCCTGGGCAACAGAGCGAGACTCTGTCTCAAAAAAATAATAATAAAAATAAAAATAAATAAAAATAAAATAACCTCAATTTCACTCTT

AGCAACACATACACACACTTAAAAACCAAGAACCACCACTGAAGTTAGGCAAGAATGGGGGAGGGGAGCTGATATAAAATAGTGATGTG

TAAGTGCACTCTACCAAGGAAAGATGATAACAATAAGCAGACTCC
```
